# Does air pollution fuel irrational behaviors in stock investments?

**DOI:** 10.1371/journal.pone.0304553

**Published:** 2024-06-06

**Authors:** Binbo Zheng, Xinbo Lu, Chih-Chun Kung, Lulu Zeng, Ping Yu

**Affiliations:** 1 School of Economics, Jiangxi University of Finance and Economics, Nanchang, China; 2 School of Public Finance and Taxtion, Zhejiang University of Finance and Economics Dongfang College, Jiaxing, China; 3 School of Economics and Center for Economic Behavior & Decision-Making (CEBD), Zhejiang University of Finance and Economics, Hangzhou, China; 4 Institute of Interdisciplinary Research, Shandong University, Weihai, China; Ben-Gurion University of the Negev, ISRAEL

## Abstract

This paper investigates the influence of air pollution on irrational behaviors in stock trading through behavioral experiments in laboratory, simulating air pollution by burning straw and mosquito coils. The results of this study show that air pollution significantly improves disposition effect and repurchase effect in an asymmetric way, which are thought as irrational behaviors in stock investments, making subjects prefer selling winning stocks (part of disposition effect) and repurchasing stocks that have fallen in price since the sale (part of repurchase effect). Furthermore, regret, a negative emotion, is the psychological mechanism by which air pollution influences the irrational behaviors.

## 1. Introduction

With the acceleration of global urbanization and industrialization, air pollution, as an unavoidable and important environmental problem, has attracted increasing attention from all sectors of society. As air pollution has an impact on all aspects of people’s lives, it becomes increasingly important to assess the cost of air pollution to society. Existing studies on the impact of air pollution on people’s physical health report that air pollution causes respiratory diseases such as pneumonia, pleurisy, bronchitis, and asthma [1–3], affects vascular function [4, 5], and shortens the human lifespan and increases mortality [6–11]. In addition, air pollution has negative impacts on neurological, cognitive, psychological, and emotional aspects, such as inducing brain inflammation or degeneration, affecting central nervous system health [12–14], and influencing the cognitive capacity of the human brain [15, 16]. Some studies have found that air pollution worsen subjects’ performance in experimental tasks [17–24]. Air pollution also affects people’s emotional and psychological well-being [25, 26] by increasing the risks of anxiety [27–30], depression [31–36], and annoyance [37]. Air pollution affects not only people’s physical and mental health but also their daily behavioral decisions. Studies have shown that air pollution influences individuals’ investment and trading decisions, causing decision bias and thus impacting investment returns [38, 39].

By simulating air pollution through laboratory experiments, this study investigated the impact of air pollution on stock investment decisions, focusing on the influence of air pollution on investors’ irrational behavior (disposition effect and repurchase effect). The "disposition effect" refers to the tendency of investors to sell winning stocks and hold losing stocks [40]. The "repurchase effect" refers to the situation when investors repurchase stocks they held before, they are more likely to repurchase stocks that have fallen in price since the sale than stocks that have increased in price since the sale [41]. According to behavioral finance, both the disposition effect and the repurchase effect are irrational behaviors of stock investors and are "financial anomalies" that are contrary to traditional financial theory. Traditional financial theory posits that stock investors’ trading decisions should be based on the judgment of future asset prices and that buying and selling prices that have occurred are sunk costs, which do not affect future decisions. In addition, it has been shown that those assets that are profitable are often more likely to continue to be profitable and that the average future one-year return of profitable stocks sold by investors is 3.4% higher than that of loss-making stocks that continue to be held [42]. The subsequent hypothesis of this study is that air pollution may trigger investors’ irrational trading decisions by affecting their emotions, which are reflected as regret in the disposition effect and repurchase effect.

Psychological explanations for the disposition effect were earlier attributed to investors’ avoidance of regret [40]. Experimental economic studies have shown that a higher level of regret leads to a more pronounced disposition effect [43, 44], a finding that also applies to the repurchase effect [41, 45–47]. In addition, neuroscientific research has shown that the activity of the ventral striatum increases when an investor sees an increase in the price of a stock after selling it and that regret activates the brain region containing the ventral striatum, meaning that investors experience regret when faced with a stock that has increased in price after selling, thus reducing the expected utility of investors to repurchase the stock [48]. Previous studies have demonstrated that the common psychological mechanism of investors’ disposition effect and repurchase effect behaviors is the triggering of regret [43, 44, 47–49].

So in this study, we hypothesized that possible mechanisms of air pollution contributing to disposition effect and repurchase effect was air pollution enhanced investors’ regret, a negative emotion, so they were prone to sell winning stocks or repurchase price-fallen stocks, and they could gain positive utility to offset negative feeling from regret. Realizing gains and repurchasing cheaper stocks are just like shopping or exercising when people feel unhappy, as ways to regulate and balance emotions.

In this study, air pollution was simulated by burning straw and mosquito coils to investigate the influence of air pollution on disposition effect and repurchase effect of stock investors through one single laboratory experiments. We believe that disposition effect and repurchase effect are two sides of the same coin, which can be examined together. This paper enriches the research on the negative effects of air pollution on irrational behaviors. In addition, the subjects provided self-reports of their regret during stock trading in the experimental task to investigate whether air pollution affects traders’ disposition effect and repurchase effect through the psychological mechanism of regret, which is difficult to be captured in non-experimental settings. The remainder of the article is organized as follows: Section 2 describes the experimental design; the experimental data are analyzed in Section 3; Section 4 focuses on the influence mechanism analysis; and research conclusions are drawn in Section 5.

## 2. Methods

### 2.1. Laboratory simulation of environmental pollution

Thus far, there has been no laboratory studies on how air pollution affects people’s economic decision-making behavior. Nevertheless, some scholars have studied the physical, psychological, and cognitive impacts of air pollution on people through laboratory experiments. Horvath et al. [50] investigated whether carbon monoxide gas is a factor in men’s reduced alertness in urban traffic driving by exposing ten subjects to carbon monoxide levels for two hours in different settings. Rotton et al. [51] studied the impact of air pollution on strangers’ ratings by using chemicals such as ammonium sulfide and butyric acid to simulate air pollution in the laboratory. Rotton et al. [52] used chemicals that are nontoxic but have an unpleasant odor—ethyl mercaptan and ammonium sulfide—to simulate air pollution and explored the impact of air pollution on conflict through laboratory experiments. Rotton [53] exposed subjects to a laboratory with a foul-smelling chemical, ethyl mercaptan, to examine the impact of air pollution on judgments and perceptions. Amitai et al. [54] exposed college students in Hebrew to different levels of carbon monoxide and tested various psychological dimensions of these students, thereby examining the impacts of air pollution on learning, attention, and visual processing abilities. Crüts et al. [12] used a double-blind randomized crossover design to expose volunteers to diluted diesel exhaust for one hour and then monitored the brain activity of these subjects by quantitative electroencephalography, finding an impact of diesel exhaust exposure on human brain function. Pope et al. [4] investigated the impact of air pollution on vascular function by exposing subjects to fine particulate matter generated by coal and wood combustion.

Drawing on the existing experimental research on air pollution, this study simulated air pollution by burning natural straw and well-known brand mosquito coils commonly available on the market to fill the air with fine particles and odors through laboratory experiments, to examine the impact of air pollution on investment decision-making behavior—disposition effect and repurchase effect. Rotton et al. [52] noted that short-term exposure to air pollution has a limited impact on human health and that air pollution can be studied in a controlled environment that does not endanger human health. In this experiment, subjects were exposed to a simulated air pollution laboratory to make stock trading decisions for an experimental duration of one hour. Although the simulated air pollution environment had limited impacts on the subjects’ life and health, it is clear from the above-mentioned laboratory studies of air pollution that the subjects’ psychological emotions and decision-making behavior could be significantly affected. Therefore, the experimental design in this study ensured that neither the subjects’ physical health nor the experimental expectations and results were affected. The protocol was approved by the Zhejiang University of Finance and Economics ethics committee.

### 2.2. Subjects

We recruited a total of 115 subjects, including 56 males and 59 females. Most of them were undergraduates, with a small number of graduate students. Their ages ranged from 18 to 26 years, with an average age of 20 years. Before the start of the experiment, all subjects signed an informed consent form to express their knowledge of air pollution (burning straw or mosquito coils) during the experiment. The experiment lasted approximately for one hour, and the average fee for participation was 70 RMB yuan. We did not receive any feedback from the subjects about any physical discomfort during or after the experiment. We conducted our experiment from November 10,2022 to November 12,2022.

### 2.3. Stock trading experiment

The design and data of our experimental task are modified from Frydman et al. [48, 49] and Li et al. [47]. This experiment is a 37-period stock investment and trading task. In the experiment, 3 stocks (A, B, C) can be traded. The computer randomly selected one stock in each period to update the price, and the price of the other two stocks remained unchanged from the previous period. Only the stock whose price was updated could be bought or sold in each period, and the other two stocks that were not displayed could not be bought or sold.

Everyone’s initial endowment was 350 experimental currency units (ECUs). Before the experiment, the system automatically helped the subjects buy 1 share of the three stocks, and their original prices were set to100 ECUs. Therefore, at the beginning of trading, every subject had 1 share of each A, B, and C and 50 ECUs. In this way, all the purchase decision in the trading would be a repurchase behavior. Everyone could hold at most one shared of each stock. For example, when the subject did not have a share of A, the computer asked him or her whether to buy this stock. When the subject held a share of A, the computer asked him or her whether to sell this stock. Short selling was not allowed in any period. We allowed the subjects to trade under debt to avoid liquidity constraints, ensuring that they had enough ECUs to buy stocks. This amount of debt was ultimately deducted from the subject’s assets. Actually, nobody was in debt at the end of the experiment or incapable of purchasing stocks due to insufficient ECUs due to large initial assets and a holding limit.

From period 1 to period 36, every subject was asked to complete the following two tasks:

Task 1: When you see the price change of a stock in this period on the computer, please report whether you regret your last trading behavior.

Task 2: Given your current stock account information, please make a trading decision (sell or repurchase the stock displayed at this period) based on this information.

The state of each stock changed over time in the following way. The price path of each stock was governed by a hidden-state Markov process with a good state and a bad state. The initial state of each stock was randomly determined (50% good state/50% bad state). Suppose that in period T, when the updated stock i is in a good state, the price of this stock had a 60% probability of rising and a 40% probability of falling in this period. In contrast, in period T, when the updated stock i was in a bad state, the price of the stock in this period had a 40% probability of rising and a 60% probability of falling.

The magnitude of the stock price increases and decreases obeyed the independent and uniform distribution of {5 ECU, 10 ECU, 15 ECU}.

If the stock price update in period T was in a good state, the probability of maintaining a good state in the next period (T+1) was 80%, and the probability of changing to a bad state was 20%. If the stock price update in period T was in a bad state, the probability of maintaining a bad state in the next period (T+1) was 80%, and the probability of changing to a good state was 20%. Nobody knew the state of the stock in the experiment, but the subjects could make Bayesian inferences about the state from observed price changes.

We used the same price path for each participant to decrease bias when comparing variables among them [48, 55]. Giving the same price path for each participant has its own weakness, that is, it is unable to avoid order effects, but the strength is also obvious. As far as we are concerned, having less bias is more important than avoiding potential order effects. To avoid the leakage of information about the price path, we added a 37th period. In this period, the computer updated all the prices of the three stocks randomly, and the final price shown to the subjects varied.

**Fig 1:**
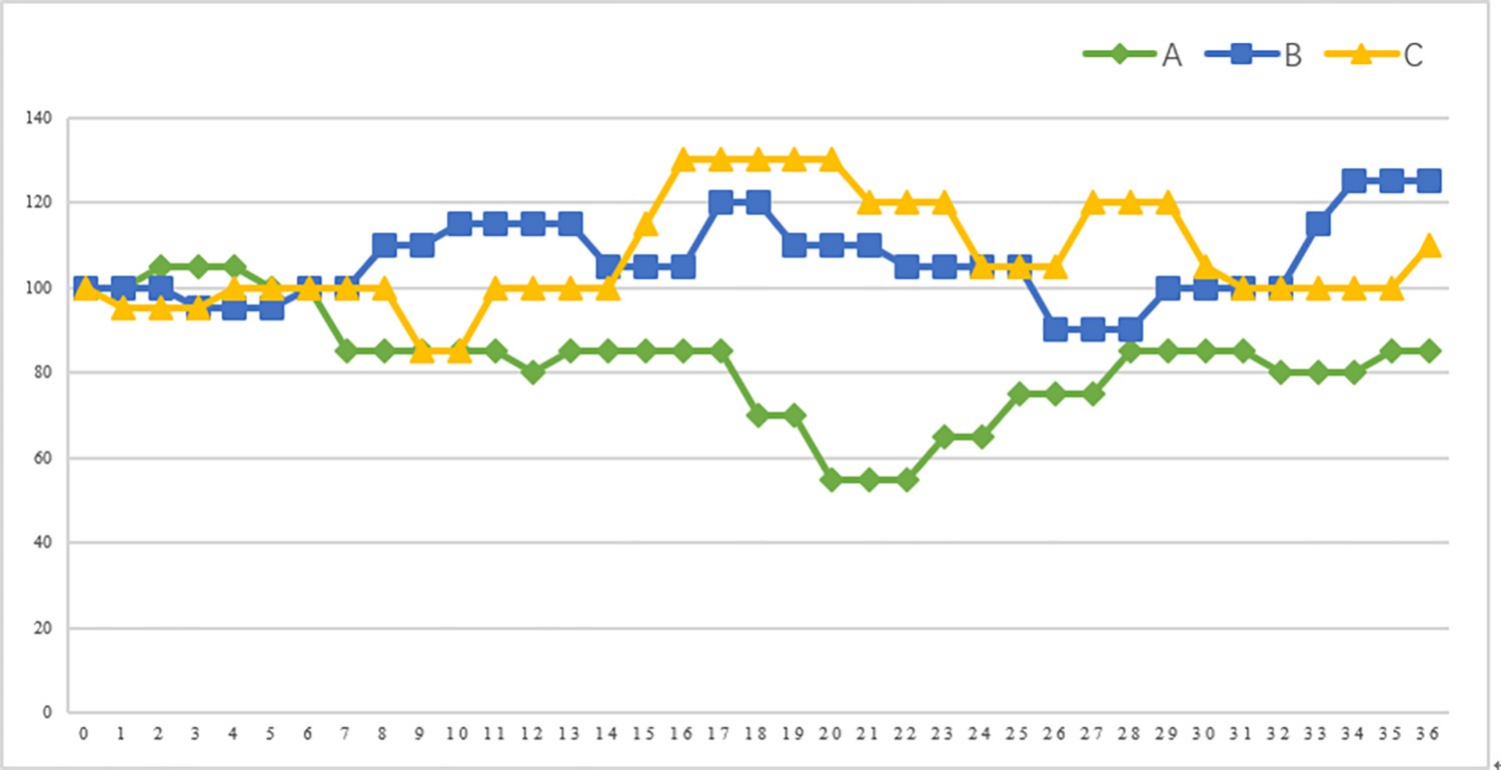
Trend path of three stocks.

The price trend path of each stock in the experiments of Frydman et al. [48, 49] and Li et al. [47] was designed with autocorrelation. The experimental rules showed that if a stock’s price increases in a certain period, it is more likely to be in a good state, then the probability of maintaining the good state in its next period will be higher, which means price increasing more likely in its next period; if a stock’s price decreases in a certain period, it is more likely to be in a bad state, then the probability of maintaining the bad state in its next period will be higher, which means price decreasing more likely in its next period. Therefore, if the subject holds the stock, the rational strategy is continuing holding it when the stock’s price rises and sell it when the stock’s price falls, which is the opposite of disposition effect. If the subject does not own the stock, the rational strategy is to repurchase it when the stock’s price rises and not to repurchase it when the stock’s price falls, which is the opposite of repurchase effect. Therefore, we put the disposition effect and the repurchase effect together as irrational behaviors in stock investment. But some investors may have a belief of mean reversion, which is, stock’s price is prone to fall after rising and rise after falling, leading them to act irrationally, such as selling rising stocks or buying falling stocks. So we remind the subject of the most likely direction of the stock price change by displaying a "↑" and "↓" arrow on the computer screen to exclude the belief of mean reversion, which is similar to Li et al. [47]. Fig 1 illustrates the price trend path of three stocks in our experiments.

## 3. Analysis of the experimental results

The variables and their meanings involved in the analysis of experimental results are shown in Table 1.

**Table 1:** The variables involved in the analysis of experimental results.

Variables	Meaning	Note
Realized gains	Selling profitable stocks	0–1 variable, where 1 is selling
Realized loses	Selling losing Stocks	0–1 variable, where 1 is selling
Paper gains	Not selling profitable stocks	0–1 variable, where 1 is not selling
Paper loses	Not selling losing Stocks	0–1 variable, where 1 is not selling
PGR	Proportion of selling profitable stocks	Realized gains/(realized gains +paper gains)
PLR	Proportion of selling losing stocks	Realized loses/(realized loses +paper loses)
DISPOSITION EFFECT	Disposition effect	PGR-PLR
Repur dows	repurchase stocks that have fallen in price after being sold	0–1 variable, where 1 is repurchase
Repur ups	repurchase stocks that have risen in price after being sold	0–1 variable, where 1 is repurchase
Non-repur dows	Not repurchase stocks that have fallen in price after being sold	0–1 variable, where 1 is not repurchase
Non-repur ups	Not repurchase stocks that have risen in price after being sold	0–1 variable, where 1 is not repurchase
PDR	The proportion of stocks that have fallen in price after being sold before repurchase	Repur dows/(repur dows +non-repur dows)
PUR	The proportion of stocks that have fallen in price after being sold before repurchase	Repur ups/(repur ups +non-repur ups)
REPURCHASE EFFECT	Repurchase effect	PGR-PLR
Hint	Trading Tips	0–1 variable, where 1 is a reminder that there may be an increase
Regret	Regret	7-point Likert scale
Pollution	Air pollution	0–1 variable, 1 is polluted
Gender	Gender	0–1 variable, 1 is male
Age	Age	Years
Major	Major	0–1 variables, 1 is majoring in economics
City	Living in City or not	0–1 variable, 1 is the city
Consume	Personal monthly consumption	Yuan
Stockexp	Financial experience	0–1 variable, 1 is having financial experience

Regarding the types of irrational decision-making behaviors, this experiment involved irrational decisions of disposition effect and irrational decisions of repurchase effect. The types of disposition effect included realizedgains and paperlosses. The types of repurchase effect included repurdows and nonrepurups. A behavior is coded as “realizedgains” when subject sells the stock at a price higher than previous buying. A behavior is coded as “paperlosses” when subject keeps the stock not to sell at a price lower than previous buying. A behavior is coded as “repurdows” when subject repurchases the stock at a price lower than previous selling. A behavior is coded as “nonrepurups” when subject choose not to buy at a price higher than previous selling.

### 3.1. Frequency analysis of irrational decision-making behavior

The subjects were divided into experimental groups, i.e., 39 in the straw-burning (ST) group and 38 in the mosquito coil-burning (MC) group, and a control group, i.e., the non-pollution group without any material burned, which had 38 subjects. Table 2 presents the frequencies of irrational decision-making behaviors in three groups; the frequencies were investigated for the entire experimental task—36 periods of the stock trading. The reason for setting up two pollution groups in the experiment—the ST group and the MC group—is that although burning different materials produces fine particulate matter, raising PM2.5 levels and thus creating air pollution, there are subtle odor differences between the two materials when they are burned. Therefore, to increase the robustness of the results, two experimental groups and one control group were included in this study.

**Table 2:** Frequency analysis of irrational decision-making behavior.

Decision type	Groups	n	Frequency
Realizedgains = 1	ST group	147	0.535
	MC group	144	0.475
	Control group	118	0.386
Paperlosses = 1	ST group	457	0.932
	MC group	457	0.935
	Control group	433	0.923
Repurdows = 1	ST group	94	0.657
	MC group	95	0.693
	Control group	63	0.504
Nonrepurups = 1	ST group	267	0.848
	MC group	231	0.813
	Control group	236	0.843

As seen in Table 2, there were frequency differences between the experimental and control groups under different decision types. Specifically, there was a large difference between frequencies in both the ST and MC groups and that in the control group under the realizedgains decision type, there was a small difference between frequencies in both the ST and MC groups and that in the control group under the paperlosses decision type, there was a large difference between frequencies in both the ST and MC groups and that in the control group under the repurdows decision type, and there was a small difference between frequencies in both the ST and MC groups and that in the control group under the nonrepurups decision type. Overall, there was a large difference in frequency between the experimental and control groups under the realizedgains decision type in the disposition effect, and there was a large difference in frequency between the experimental and control groups under the repurdows decision type in the repurchase effect. Therefore, air pollution had an impact on realizedgains decision-making behavior in the disposition effect and an impact on repurdows decision-making behavior in the repurchase effect.

### 3.2. Mann‒Whitney test of irrational decision types

In terms of experimental design, the aim of this study was to determine whether environmental pollution influenced the subjects’ irrational decision-making behaviors in a stock investment experimental task. Therefore, the differences between the pollution group (ST group or MC group) and the control group were comparatively analyzed. STATA software are used to statistically evaluate all our experimental data. We set p < 0.05 for the critical level of significance for the Mann‒Whitney test.

As seen in Table 3, for the experiment in which air pollution was simulated by burning straw, there was a significant difference between the ST group and the control group under the realizedgains decision type (Mann‒Whitney test, z = -3.595, p = 0.0003), no significant difference under the paperlosses decision type (Mann‒Whitney test, *z* = -0.564, p = 0.5731), a significant difference under the repurdows decision type (Mann‒Whitney test, *z* = -2.538, p = 0.0112), and no significant difference under the nonrepurups decision type (Mann‒Whitney test, *z* = -0.160, p = 0.8727). Therefore, compared with no pollution, the air pollution simulated by burning straw significantly influenced the subjects’ irrational decision-making behaviors regarding realizedgains and repurdows in the stock trading experiment.

**Table 3:** Mann‒Whitney test of the irrational decision types between the ST group and control group.

Variables	Groups	Ranksum	Obs.	Rankmean	z	p
Realizedgains	ST group	86291	275	313.79	-3.595	0.000
	Control group	82780	306	270.52		
Paperlosses	ST group	236281.5	490	482.21	-0.564	0.573
	Control group	224038.5	469	477.69		
Repurdows	ST group	20604	143	144.08	-2.538	0.011
	Control group	15442	125	123.54		
Nonrepurups	ST group	94080	315	298.67	-0.160	0.873
	Control group	83230	280	297.25		

As seen in Table 4, in the experiment in which air pollution was simulated by burning mosquito coils, there was a significant difference between the MC group and the control group under the realizedgains decision type (Mann‒Whitney test, *z* = -2.232, p = 0.0256), no significant difference under the paperlosses decision type (Mann‒Whitney test, *z* = -0.682, p = 0.4955), a significant difference under the repurdows decision type (Mann‒Whitney test, *z* = -3.124, p = 0.0018), and no significant difference under the nonrepurups decision type (Mann‒Whitney test, *z* = 0.927, p = 0.3541). Therefore, compared with no pollution, the air pollution simulated by burning mosquito coils significantly influenced the subjects’ irrational decision-making behaviors regarding realizedgains and repurdows in the stock trading experiment.

**Table 4:** Mann‒Whitney test of the irrational decision types between the MC group and control group.

Variables	Groups	Ranksum	Obs.	Rankmean	z	p
Realizedgains	MC group	96570	303	318.71	-2.232	0.026
	Control group	89175	306	291.42		
Paperlosses	MC group	235773.5	489	482.15	-0.682	0.496
	Control group	223587.5	469	476.73		
Repurdows	MC group	19637.5	137	143.34	-3.124	0.002
	Control group	14815.5	125	118.52		
Nonrepurups	MC group	79058	284	278.37	0.927	0.354
	Control group	80272	280	286.69		

As seen in Tables 3 and 4, burning straw and mosquito coils to simulate air pollution significantly influenced the subjects’ irrational decision-making behaviors regarding realizedgains and repurdows in the stock trading experiment.

### 3.3. Mann‒Whitney test of the disposition effect and repurchase effect

In order to investigate the impact of air pollution on irrational behaviors with more details, we measure the disposition effect and repurchase effect in another way. The measure of the disposition effect is computed based on the method developed by Odean [42]. “DISPOSITION” is computed as the difference between PGR (proportion of gains realized) and PLR (proportion of losses realized). The measure of the repurchase effect is computed based on the method developed by Strahilevitz et al. [41]. “REPURCHASE” equals PDR (proportion of stocks repurchased after prices decrease) minus PUR (proportion of stocks repurchased after prices increase).

**Fig 2:**
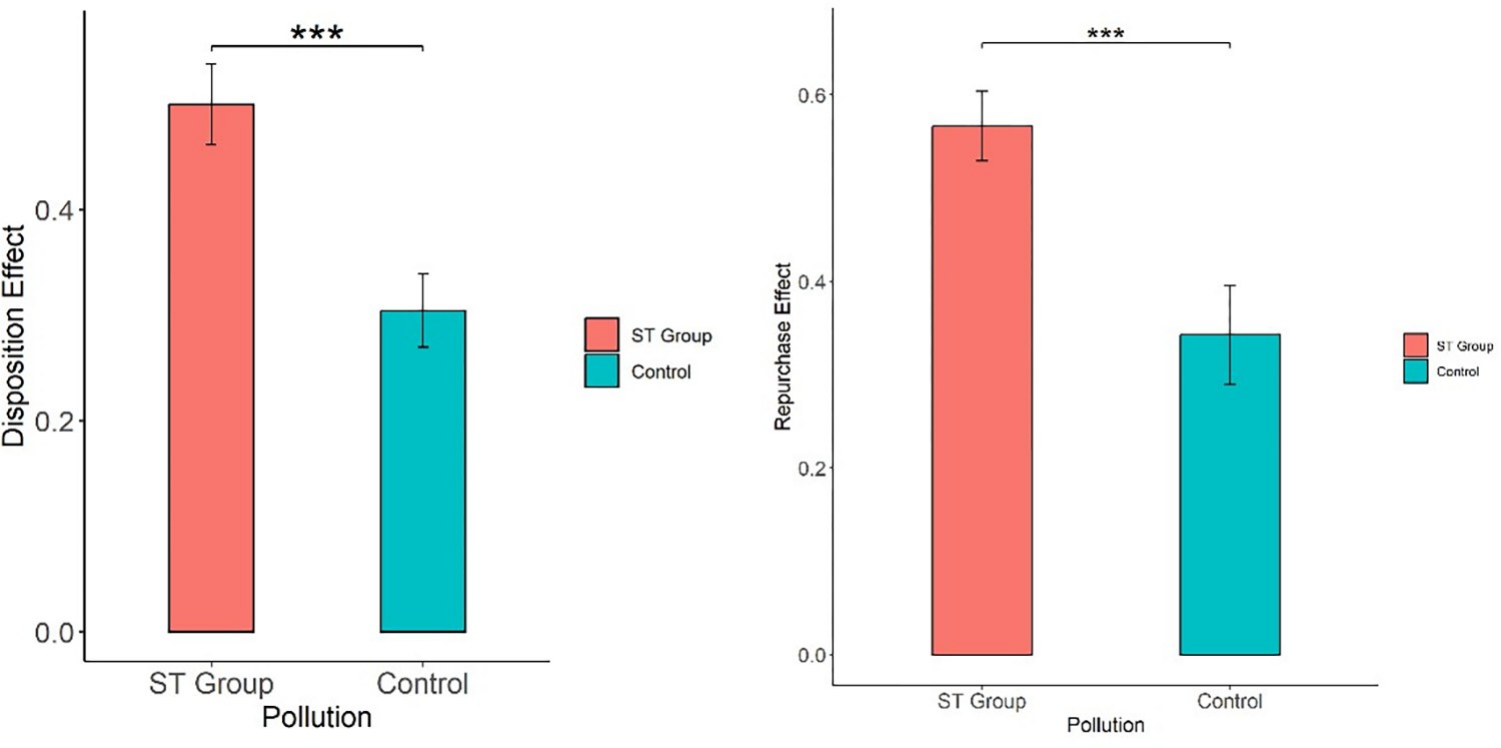
Impact of ST air pollution on disposition effect and repurchase effect.

As seen in Table 5, in the experiment in which air pollution was simulated by burning straw, there was a significant difference between the ST group and the control group under PGR (Mann‒Whitney test, *z* = -3.559, p = 0.0004), no significant difference under PLR (Mann‒Whitney test, *z* = 0.526, p = 0.5986), a significant difference under DISPOSITION (Mann‒Whitney test, *z* = -3.536, p = 0.0004), a significant difference under PDR (Mann‒Whitney test, *z* = -2.642, p = 0.0082), no significant difference under PUR (Mann‒Whitney test, *z* = 1.247, p = 0.2125), and a significant difference under REPURCHASE (Mann‒Whitney test, *z* = -3.313, p = 0.0009). Fig 2 also illustrated the result of disposition effect and repurchase effect between ST group and control group. Therefore, compared with no pollution, the air pollution simulated by burning straw significantly influenced the subjects’ disposition effect and repurchase effect in the stock trading experiment. The influence on the disposition effect and the repurchase effect was asymmetric; that is, air pollution only influenced PGR under the disposition effect and PDR under the repurchase effect.

**Table 5:** Mann‒Whitney test of the disposition effect and repurchase effect between the ST group and control group.

Variables	Groups	Ranksum	Obs.	Rankmean	z	p
PGR	ST group	1869.5	39	47.94	-3.559	0.000
	Control group	1133.5	38	29.83		
PLR	ST group	1470.5	39	37.71	0.526	0.599
	Control group	1532.5	38	40.33		
DISPOSITION	ST group	1868	39	47.90	-3.536	0.000
	Control group	1135	38	29.87		
PDR	ST group	1689.5	38	44.46	-2.642	0.008
	Control group	1160.5	37	31.36		
PUR	ST group	1399.5	39	35.88	1.247	0.213
	Control group	1603.5	38	42.20		
REPURCHASE	ST group	1756.5	38	46.22	-3.313	0.001
	Control group	1093.5	37	29.55		

**Fig 3:**
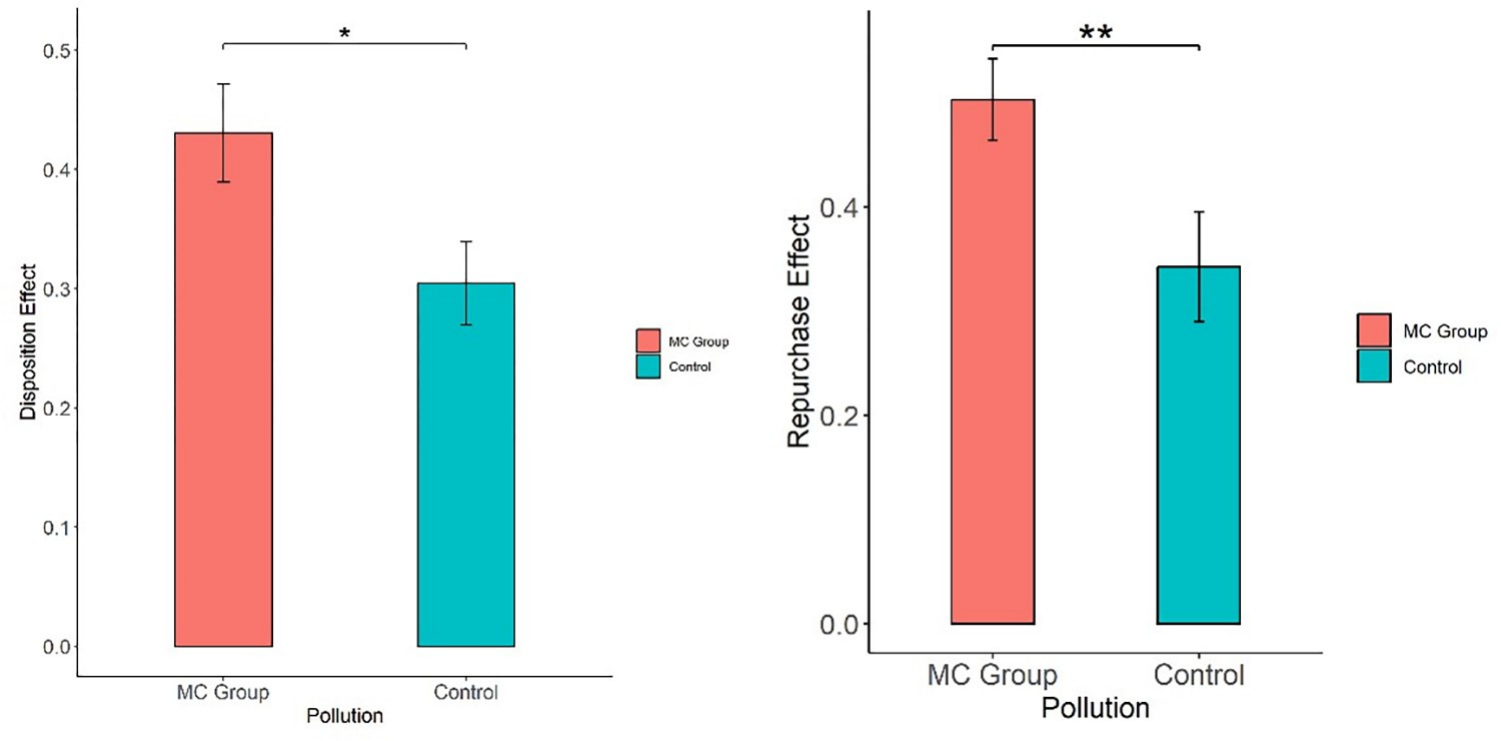
Impact of MC air pollution on disposition effect and repurchase effect.

As seen in Table 6, in the experiment in which air pollution was simulated by burning mosquito coils, there was a significant difference between the MC group and the control group under PGR (Mann‒Whitney test, *z* = -2.049, p = 0.0404), no significant difference under PLR (Mann‒Whitney test, *z* = 0.624, p = 0.5326), no significant difference under DISPOSITION (Mann‒Whitney test, *z* = -1.917, p = 0.0552), a significant difference under PDR (Mann‒Whitney test, *z* = -2.831, p = 0.0046), no significant difference under PUR (Mann‒Whitney test, *z* = -0.893, p = 0.3717), and a significant difference under REPURCHASE (Mann‒Whitney test, *z* = -2.445, p = 0.0145). Fig 3 also illustrated the result of disposition effect and repurchase effect between MC group and control group. Therefore, compared with no pollution, the air pollution simulated by burning mosquito coils significantly influenced the subjects’ repurchase effect in the stock trading experiment. The influence on the disposition effect and the repurchase effect was asymmetrical; that is, air pollution only influenced PGR under the disposition effect and PDR under the repurchase effect.

**Table 6:** Mann‒Whitney test of the disposition effect and repurchase effect between the MC group and control group.

Variables	Groups	Ranksum	Obs.	Rankmean	z	p
PGR	MC group	1660	38	43.68	-2.049	0.040
	Control group	1266	38	33.32		
PLR	MC group	1404.5	38	36.96	0.624	0.533
	Control group	1521.5	38	40.04		
DISPOSITION	MC group	1647.5	38	43.36	-1.917	0.055
	Control group	1278.5	38	33.64		
PDR	MC group	1707.5	38	44.93	-2.831	0.005
	Control group	1142.5	37	30.88		
PUR	MC group	1548.5	38	40.75	-0.893	0.372
	Control group	1377.5	38	36.25		
REPURCHASE	MC group	1674.5	38	44.07	-2.445	0.015
	Control group	1175.5	37	31.77		

In summary, the air pollution simulated by burning straw significantly influenced the subjects’ disposition effect and repurchase effect in the stock experiment, and the air pollution simulated by burning mosquito coils significantly influenced the subjects’ repurchase effect in the stock experiment. Notably, the air pollution simulated by burning each material had asymmetric influences on the disposition effect and the repurchase effect; that is, the air pollution simulated by burning each material only influenced PGR under the disposition effect and PDR under the repurchase effect.

## 4. Discussion: Analysis of the influence mechanism

### 4.1. Analysis of the influence mechanism

**Fig 4:**
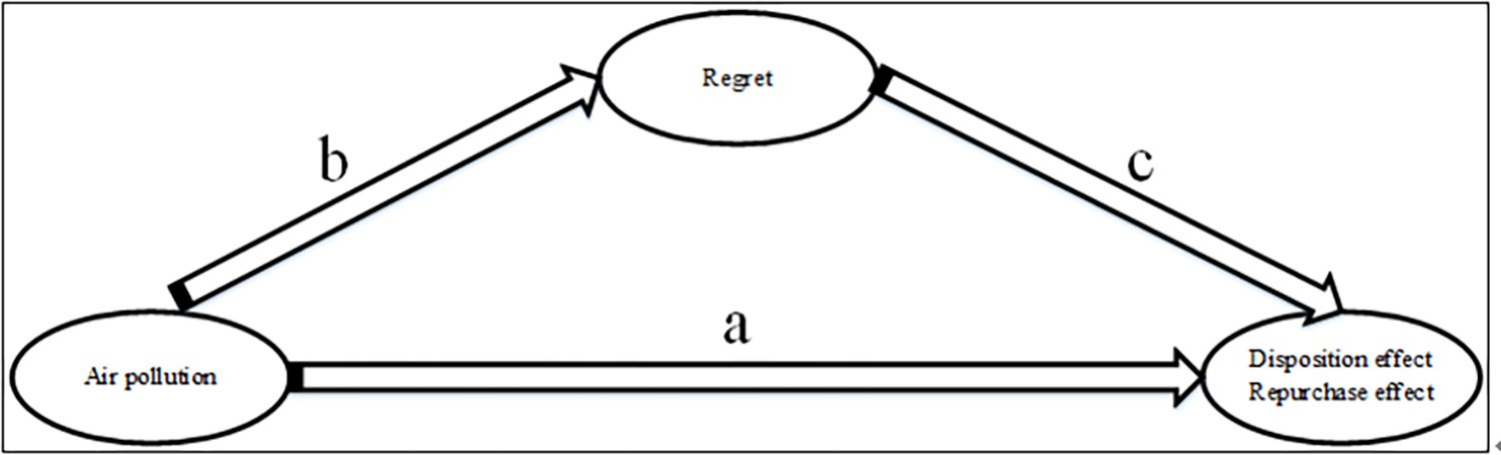
Influence path about air pollution, regret and disposition effect/ repurchase effect.

Air pollution can cause investors’ irrational behavior in the stock market—the disposition effect. The bias of this irrational behavior mainly stems from people’s negative emotional feedback, for example, regret. This study argues that this influence mechanism also applies to the repurchase effect. Fig 4 illustrates the influence path of this mechanism.

According to Gintis [56], the analysis of behavioral economics is mainly based on the BPC model, i.e., "beliefs", "preferences", and "constraints". The BPC model holds that human behavior is a process of maximizing one’s own preferences given constraints and beliefs and that such preferences should conditionally conform to the requirements of the consistency axiom—completeness, transitivity, and independence of irrelevant choices; that is, preferences should be context dependent.

The emergence of modern psychology in the 20th century has made people gradually realize that the influence of individuals’ psychology on their behavior can spill over into market performance. People’s economic behavior is often influenced by their psychological state in the face of economic risks and uncertainties in the market. Regret is generally based on people’s perception of negative emotions, and it is commonly accepted in Psychology that regret is a complex and compound emotional experience that is distinct from basic emotions such as joy, anger, sadness, and sorrow and comes from counterfactual thinking, a deep-level cognitive processing in the brain. The experience of regret implies that individuals have the ability to envision the current outcome as well as other possible outcomes; the results reported by Guttentag and Ferrell [57] confirm this conjecture.

Behavioral economics research has shown that when people are faced with choices, they seek to not only maximize utility but also minimize regret afterward [58–62]. The most common emotion in human decision-making behavior is regret; that is, when there are multiple options, people easily fall into choice difficulty, and this situation occurs because people may regret afterward that the unchosen option would have been better than the chosen one. A common example of regret that causes decision-making bias in economic behavior is the disposition effect in stock trading.

Research on the disposition effect in stock trading have mainly been based on the observation and discussion of financial market anomalies. Some scholars have discussed the disposition effect in stock investment through an empirical and statistical approach to reveal the psychological or cognitive biases behind this anomaly [40, 42, 63, 64]. Other scholars have focused on experimental methods, which are more intuitive than empirical methods for behavioral research, and designed relevant experiments to identify subjects’ behavioral biases from their performance in experimental tasks [47–49].

Shefrin and Statman [40] introduced the concept of "regret aversion" in 1985 to explain why investors sell winning stocks and hold losing stocks for a long period of time. They argued that investors often experience this repetitive behavior in the investment process: when people make bad investment decisions, even small mistakes, they experience regret and feel very remorseful, instead of taking a long-term view of these mistakes or taking measures to avoid making the same financial decision mistakes again. When the stock market is in a bull market, people often regret that they did not purchase stocks they liked or sold profitable stocks too early; in contrast, when the stock market is in a bear market, investors often regret that they failed to stop their losses quickly, or they may regret that the stocks they held did not rise while the stocks that others recommended to them but they did not purchase did increase, thus missing an opportunity to profit. Therefore, when investors make financial decisions, to avoid this regret or delay the perception of this regret, they engage in irrational behavior—the disposition effect.

The negative emotion of regret proposed in this study caused the subjects’ behavioral biases, such as the disposition effect and repurchase effect in stock trading, which primarily arose from changes in the individual preferences of the subjects; such changes are actually context-dependent preferences according to behavioral economists. As the subjects made stock investment decisions in different contexts, their stock preferences changed accordingly, an effect mainly attributed to the susceptibility of the subjects to the feedback of regret as a negative emotion. In addition, Kajol et al. [65] used social network analysis to analyze the factors influencing the disposition effect of stock investors and found that social trust and investor sentiment are the two most important factors influencing the disposition effect.

Regarding the influence paths *a* and *c* in Fig 4, we have discussed path *a*, that is, air pollution influences the disposition effect in stock trading, in the Introduction section. In this section, we discuss path *c*, that is, the influence of regret on the disposition effect. Studies have found that air pollution can cause negative emotions in people. Mehrabian & Russell [66] reported that air pollution affects people’s cognitive or emotional state. Li et al. [39] found that air pollution influences the effectiveness of trading decisions and trading behaviors in financial markets by affecting individual investors’ psychological, emotional, and cognitive abilities, thus increasing the likelihood of making mistakes such as the disposition effect; they showed that hazy weather increases the disposition effect of individual investors and argued that haze may influence individuals’ investment decision-making ability and the effectiveness of financial markets by affecting investors’ emotional or cognitive abilities.

The main reason for considering regret as a psychological mechanism by which air pollution affects disposition effect behavior is that, as found in previous studies, the stock disposition effect is more closely related to investors’ regret than to other negative emotions, and regret is an important psychological factor influencing investors’ disposition effect behavior. In addition, in the experimental task of stock trading, the subjects provided self-reports of their regret during the trading process. The data analysis revealed that there was a significant difference in the reported regret between the experimental and control groups, indicating that air pollution affected the subjects’ regret perceptions in this experiment.

**Fig 5:**
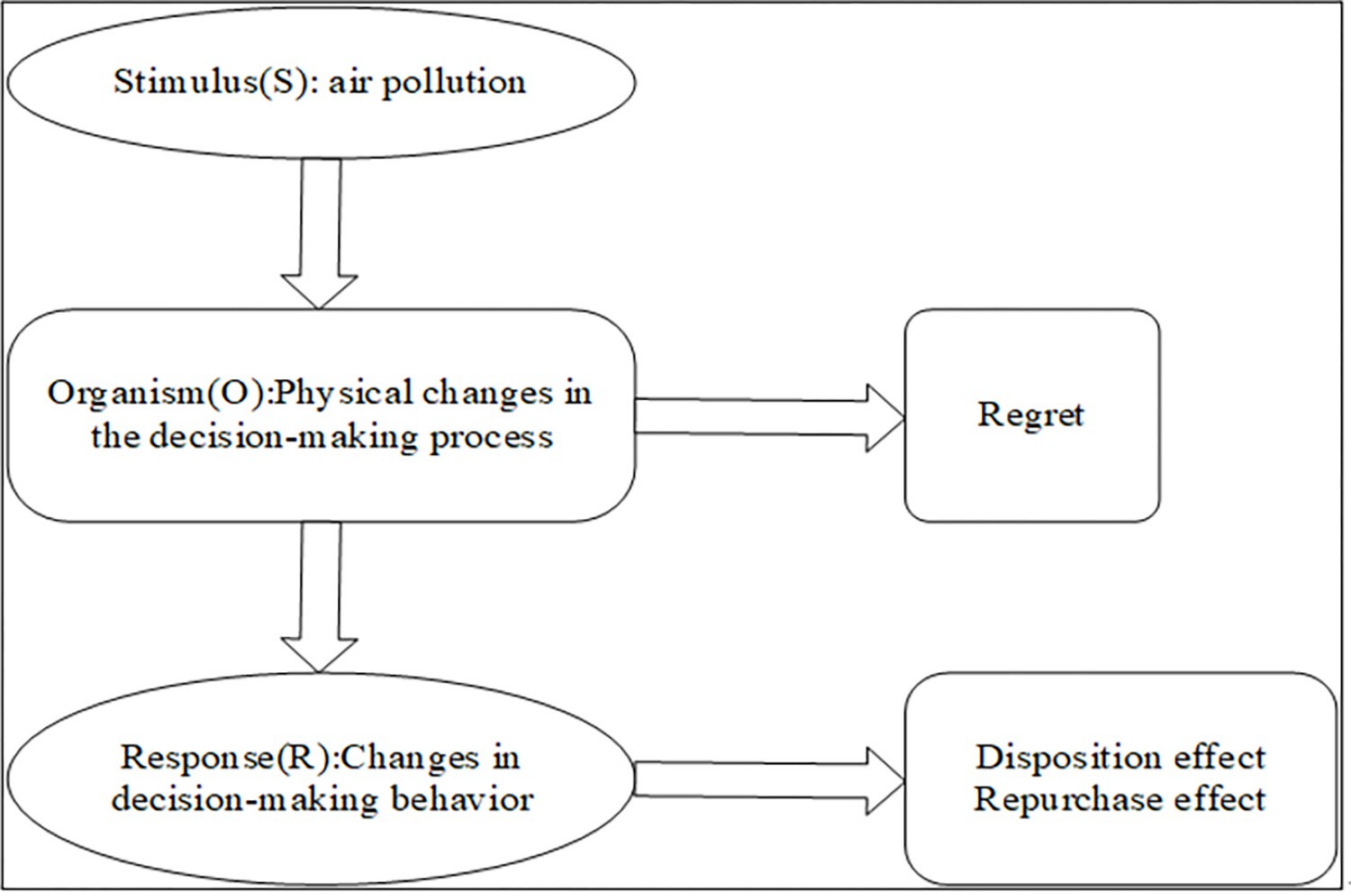
Influence mechanism framework based on stimulus-organism-response (S-O-R).

We noted above that the negative feedback of regret is the psychological mechanism by which air pollution influences the stock disposition effect. In the stock market, the buying and selling roles of stock investors change at any time. Because of these changes in roles, the principles and mechanisms of the disposition effect and the repurchase effect faced by investors are the same. Therefore, we believe that the above analysis is equally applicable to the analysis of the repurchase effect behavior of investors. On the basis of the above analysis, an influence mechanism framework is established based on stimulus-organism-response (S-O-R) in Fig 5. In this framework, air pollution, as an exogenous stimulus, affects the changes in stock traders’ trading decision-making behavior by influencing their physiological changes (i.e., regret perceptions) during the trading process, thereby leading to irrational behaviors such as the disposition effect and repurchase effect.

### 4.2. Verification with data of path a

As seen in Table 7, after controlling for gender, age, profession, city, individual monthly consumption, and whether subjects had financial management experience, the influence of air pollution on the disposition effect and repurchase effect is significant at the 1% significance, and the coefficients are positive, thus indicating that air pollution has a positive impact on the disposition effect and the repurchase effect. In addition, the influence of air pollution on PGR is significant at the 1% level, and the coefficient is positive; furthermore, the influence of air pollution on PDR is significant at the 5% level, and the coefficient is positive. The influence on PLR and PUR is not significant, further indicating that there is a positive impact of air pollution on PGR and PDR; that is, the influence of air pollution on the disposition effect and repurchase effect are asymmetric. Therefore, air pollution significantly increased the subjects’ irrational decision-making behavior in stock investment, including PGR in the disposition effect and PDR in the repurchase effect.

**Table 7:** OLS regression of air pollution and the disposition effect and repurchase effect.

Variables	PGR	PLR	DISPOSITION	PDR	PUR	REPURCHASE
Pollution	0.135***	-0.003	0.138***	0.148**	-0.005	0.152***
	(0.045)	(0.017)	(0.049)	(0.057)	(0.023)	(0.057)
Gender	-0.074*	0.009	-0.083*	0.020	0.025	-0.005
	(0.042)	(0.016)	(0.045)	(0.053)	(0.021)	(0.052)
Age	-0.007	0.002	-0.009	-0.03**	-0.011	-0.028*
	(0.013)	(0.005)	(0.014)	(0.017)	(0.007)	(0.016)
Major	0.039	0.009	0.030	0.024	-0.012	0.035
	(0.051)	(0.019)	(0.055)	(0.064)	(0.026)	(0.063)
City	-0.001	0.013	-0.014	-0.018	-0.005	-0.012
	(0.042)	(0.016)	(0.046)	(0.053)	(0.022)	(0.052)
Consume	0.034	-0.023***	0.057**	0.022	-0.024**	0.046
	(0.023)	(0.009)	(0.025)	(0.029)	(0.012)	(0.028)
Stockexp	-0.012	-0.018	0.006	0.006	0.010	-0.007
	(0.056)	(0.022)	(0.061)	(0.073)	(0.029)	(0.072)
Constant	0.445	0.100	0.344	1.209**	0.442***	0.762**
	(0.272)	(0.103)	(0.294)	(0.343)	(0.139)	(0.338)
Obs	115	115	115	113	115	113
R^2^	0.150	0.077	0.167	0.153	0.071	0.153

NOTE

***p < 0.01

**p < 0.05

*p < 0.1

### 4.3. Verification with data of path b

We measured individual differences in regret before the start of the experiment and measured individuals’ baseline regret using the scenario designed by Kahneman & Tversky [67]. The results showed that there was no significant difference in the regret of individuals in the experimental and control groups. Therefore, fluctuations in regret for different subjects in the experimental task were all caused by the influence of the laboratory environment. As seen in Table 8, the positive influence of air pollution on regret significantly exists at the 1% level, indicating air pollution was more likely to trigger the subjects’ regret in decision-making behavior.

**Table 8:** Mann‒Whitney test of regret between the pollution group and control group.

Variable	Groups	Ranksum	Obs.	Rankmean	z	p
Regret	Pollution group	5848752.5	2772	2109.9	-3.125	0.0018
	Control group	2723117.5	1368	1990.6		

### 4.4. Verification with data of path c

As seen in Table 9, the regressions for regret and realizedgains and paperlosses are both significant at the 1% level, and the coefficients are positive; the regression for repurdows is significant at the 5% level, and the coefficient is positive; and the regression for nonrepurups is significant at the 1% level, and the coefficient is negative. Based on the results of the earlier analysis, the influence of air pollution on irrational decision-making behaviors is asymmetric; that is, air pollution significantly influenced realizedgains and repurdows as two irrational decision-making behaviors. Therefore, here, we only examined the influence of regret on realizedgains and repurdows, and the results indicated that regret had a positive impact on both realizedgains and repurdows; that is, regret increased the subjects’ irrational behaviors in the stock trading task, mainly realizedgains under the disposition effect and repurdows under the repurchase effect.

**Table 9:** OLS regression of regret and irrational decision types.

Variables	Realizedgains	Repurdows
Regret	0.0477***	0.0536**
	(0.0141)	(0.0209)
Hint	0.184***	0.0477
	(0.0340)	(0.0488)
Constant	0.344***	0.548***
	(0.0258)	(0.0366)
Obs	884	405
R-squared	0.0422	0.0204

NOTE

***p < 0.01

**p < 0.05

*p < 0.1

## 5. Conclusion

This study simulated air pollution by burning straw and mosquito coils and investigated the influence of air pollution on irrational behaviors (disposition effect and repurchase effect) in stock trading through laboratory experiments. The experimental results showed that air pollution simulated by burning straw and mosquito coils significantly influenced the irrational decision-making behaviors (realizedgains and repurdows) of the subjects in the stock trading experiment. The air pollution simulated by burning straw significantly influenced the disposition effect and repurchase effect of the subjects in the stock trading experiment, and the air pollution simulated by burning mosquito coils significantly influenced the repurchase effect of the subjects in the stock trading experiment. Notably, the air pollution simulated by each material had asymmetric influences on the disposition effect and the repurchase effect; that is, the air pollution simulated by each material only influenced PGR under the disposition effect and PDR under the repurchase effect.

An S-O-R-based framework was established in this study to analyze the mechanism by which air pollution influences the disposition effect and repurchase effect. In the S-O-R analysis framework, air pollution, as an exogenous stimulus, affects the changes in stock traders’ trading decision-making behavior by influencing their physiological changes (i.e., regret perceptions) during the trading process, thereby leading to irrational behaviors such as the disposition effect and repurchase effect. S-O-R analysis showed that air pollution influenced the disposition effect and repurchase effect of subjects’ stock trading by affecting their regret. Path test results indicated that air pollution was more likely to induce subjects’ regret, which in turn increased their irrational decision-making behaviors in stock investments, including PGR under the disposition effect and PDR under the repurchase effect. As for the reason why we only have significant results in PGR and PDR, the possible explanation is that, selling a stock whose price is higher than buying to realize gains or repurchasing a stock whose price is lower than selling are more easily to relieve negative emotions(regret) triggered by air pollution, which can make investors think that they have "made a profit or taken advantage" to obtain inner pleasure.

This studied found that air pollution influences individuals’ emotions and thus leads to irrational decision-making through behavioral experiments. However, it could also exist other underlying mechanism not found, which would also be a limitation of this paper. But what we found is still meaningful and important in shaping investor behavior and exploring the potential mechanism, giving people an enlightenment about air pollution’s influences. Further studies could conduct more complex experiment to investigate the emotion or other potential mental or psychological characteristics of subjects in tasks. Besides, individual emotions were not investigated very accurately in this paper. Subsequent research could consider using neural experimental techniques such as fMRI and tDCS to identify the neural basis of this psychoemotional influence. In addition, our investigation of regret was based on subjective self-reports by the subjects, thus leading to limits of data in someway. Subsequent research should consider using a multi-channel electrophysiology recorder to capture physiological signals of subjects in different experimental settings, such as ECG, EEG, EMG, respiration, and blood flow, so as to more objectively explain the physiological and psychological mechanisms underlying the behavioral differences of the subjects in experimental tasks.

## Supporting information

S1 Data (XLSX)
